# Protective Effects of Interleukin-1 Blockade on Group B *Streptococcus*-Induced Chorioamnionitis and Subsequent Neurobehavioral Impairments of the Offspring

**DOI:** 10.3389/fendo.2022.833121

**Published:** 2022-07-01

**Authors:** Taghreed A. Ayash, Seline Y. Vancolen, Mariela Segura, Marie-Julie Allard, Guillaume Sebire

**Affiliations:** ^1^Department of Pediatrics, McGill University, Montreal, QC, Canada; ^2^Department of Pharmacology, McGill University, Montreal, QC, Canada; ^3^Faculty of Veterinary Medicine, Université de Montreal, St-Hyacinthe, QC, Canada

**Keywords:** autism spectrum disorder (ASD), cerebral palsy (CP), fetal inflammatory response syndrome (FIRS), hyperactivity, interleukin-1 receptor antagonist, maternal immune activation, placentoprotection, neuroplacentology

## Abstract

Group B *Streptococcus* (GBS) is one of the most common bacteria isolated in human chorioamnionitis. Placental infection due to GBS is a major risk factor for fetal organ injuries, preterm birth, perinatal morbidity and mortality, and life-long multiorgan morbidities. Preclinical and clinical studies have shown that GBS-induced infection drives polymorphonuclear (PMN) cell infiltration within the placenta, the hallmark of human chorioamnionitis. In preclinical and clinical studies, the upregulation of interleukin(IL)-1β in the placenta and maternal/fetal blood was associated with a high risk of neurodevelopmental impairments in the progeny. We hypothesized that targeted IL-1 blockade administered to the dam alleviates GBS-induced chorioamnionitis and the downstream fetal inflammatory response syndrome (FIRS). IL-1 receptor antagonist (IL-1Ra) improved the gestational weight gain of GBS-infected dams and did not worsen the infectious manifestations. IL-1Ra reduced the IL-1β titer in the maternal sera of GBS-infected dams. IL-1Ra decreased the levels of IL-1β, IL-6, chemokine (C-X-C motif) ligand 1 (CXCL1), and polymorphonuclear (PMN) infiltration in GBS-infected placenta. IL-1Ra treatment reduced the IL-1β titer in the fetal sera of GBS-exposed fetuses. IL-1 blockade also alleviated GBS-induced FIRS and subsequent neurobehavioral impairments of the offspring without worsening the outcome of GBS infection. Altogether, these results showed that IL-1 plays a key role in the physiopathology of live GBS-induced chorioamnionitis and consequent neurobehavioral impairments.

## Introduction

Epidemiological and preclinical studies linked intrauterine infections, maternofetal immune activation (MIA), and neurodevelopmental impairments in offspring (1–7). Most experimental designs aiming to investigate the impact of MIA triggered by bacteria have used lipopolysaccharide (LPS) from the Gram-negative bacillus *Escherichia coli* – which acts mainly through Toll-like receptor 4 (TLR4) (4, 7–12). Gram-positive *cocci* like Group B *Streptococcus* (GBS, *Streptococcus agalactiae*) use different inflammatory signaling pathways than LPS, such as TLR2 and TLR6 and β-hemolysin/NOD-like receptor (NLR)-P3 pathway-driven placental inflammation. This pro-inflammatory cascade induces polymorphonuclear (PMN) cell infiltration in GBS-infected tissues, as observed in human chorioamnionitis (13–16). Preclinical investigations have shown that GBS-induced inflammation is associated with neurodevelopmental impairments such as intrauterine growth retardation, cerebral palsy- (CP), hyperactivity-, and autism spectrum disorder (ASD)-like behaviors (17, 18). Elucidating the molecular mechanisms involved in GBS infection and GBS-induced MIA represents a significant health care need, as these disorders remain very prevalent in the population despite improvements in perinatal healthcare (19–22). As the nature of the infecting pathogen affects MIA, it is essential to conduct pathogen-specific studies profiling the inflammatory responses to develop personalized treatment strategies.

Administration of IL-1β in dams has been shown to induce dose-dependent functional and structural changes in placental and fetal brain tissues (23, 24). IL-1β has also been shown to be associated with the maternofetal immune responses consequent of LPS and GBS exposure *in utero* (7, 8, 25, 26). Using a preclinical rat model of GBS-induced chorioamnionitis, we established that GBS inoculated intraperitoneally (i.p.) to the dam: (*i*) infects the placenta (24), *(ii)* rapidly induces the overexpression of IL-1β in the placentas (27) *(iii)* and leads to sexually dichotomous chorioamnionitis, with higher IL-1β release and PMN recruitment in male *versus* litter-matched female tissues (24, 27).

Our laboratory and others have shown end-gestational IL-1 blockade to be placento- and neuro-protective in LPS-induced chorioamnionitis and reduce subsequent neurodevelopmental anomalies (7, 8, 11, 28). However, no study had investigated yet whether this maternal treatment could also be suitable in GBS-induced chorioamnionitis and, more broadly, in an active bacterial infection, in which IL-1 blockade might affect anti-infectious immunity.

Initially approved by the FDA in 2001, the human recombinant (hr) IL-1 receptor antagonist (IL-1Ra), anakinra, is an effective therapeutic option with a short half-life and a high safety profile already used in clinical practice for the treatment of rheumatoid arthritis and certain autoinflammatory disorders (8, 29–32). Anakinra administration has already been shown to be well tolerated during pregnancy, with satisfactory pregnancy outcomes (33–37). Anakinra has also been suggested to be a safe therapeutic option to attenuate the cytokine storm triggered by the viral infection in pregnant women with severe Covid-19 (38, 39). The aim of this study tests whether end-gestational IL-1 blockade decreases GBS-induced placental and fetal inflammation without aggravating GBS infection and prevents neurobehavioral impairments of the offspring.

## Material & Methods

### Animals

All experiments were approved by the Research Institute of McGill University Health Centre (RI-MUHC) and performed according to the Canadian Council on Animal Care. Pregnant Lewis dams (n = 23) were obtained from Charles River Laboratories (Kingston, NY, US) at gestational day (G)13. They were housed at the RI-MUHC animal facility (Glen site, Montreal, QC, Canada) in a controlled environment (20°C with 12 h light/dark cycle) and had access to water and food *ad libitum.* All injections were performed i.p. At G19, dams were randomized into three groups, namely: (1) CTL dams (number (n) of dams = 6) injected with saline, (2) GBS plus saline-inoculated dams (n = 9), and (3) GBS plus hrIL-1Ra-injected dams (n = 8). At 36, 48, and 60 h following GBS inoculation, dams from the GBS plus IL-1Ra group were injected either with 100 µL of sterile 0.9% saline, or with 100 µL of 10 mg/kg of IL-1Ra (RI0322, Neo Scientific, MA, US). The same treatment protocol of 10 mg/kg/12 h of hrIL-1Ra has been already shown to be placento-protective and well-tolerated in a rat model of LPS-induced chorioamnionitis (40).

### Bacterial Growth Conditions

A stock of β-hemolytic capsular serotype la GBS (strain #16955), stored at -80°C in brain heart infusion (BHI) broth with 15% glycerol, was used for all experiments (7, 24). Bacterial preparation was performed as previously described (24, 27). Briefly, an aliquot containing the GBS bacteria in 0.9% of sterile saline was kept on ice until the time of injection. Dilutions between 10^-5^ and 10^-10^ were plated in triplicate on BHI agar and incubated overnight at 37°C. As previously described, the targeted final GBS dose was 10^8^ colony-forming units per 100 µl (24). To rule out any contamination, each GBS dose was tested for identification on CHROMID Strepto B Agar Plate (BioMerieux, Saint-Laurent, QC, Canada).

### Gestational Monitoring, Caesarian-Section and Tissue Sampling

Dams were weighed twice per day and regularly observed to detect any sickness behavior. Maternal weight gain was calculated by subtracting the weight measured at G22 minus the weight at G18. Dams underwent C-sections at G22 (72 h post-inoculation). This time point was chosen based on previous sequential measures of the inflammatory markers in this model, showing that the placental and fetal expression peaks occurred at 72 h post-GBS i.p. inoculation (24). Maternal/fetal blood and placentas were collected, processed and stored as previously described (24). Briefly, C-sections were performed while dams remained under deep anesthesia (2% isoflurane, 1.5% O_2_). Alive fetuses were collected along with their associated placenta. The weight of each fetus and placentas was measured. Fetal and maternal blood were collected in Lithium Heparin Gel Separator tubes (BD Microtainer blood collection tubes, BD, NJ, US), then centrifuged, aliquoted and stored at -80°C until analysis. The number of dead and alive fetuses was used to determine the mortality rate per litter. Placentas were separated from the fetuses and cut on the median coronal plan (23, 24).

### Histopathology and Immunohistochemistry

Fixed samples were processed, paraffin-embedded, and two adjacent 5-μm thick sections were used per slide (VWR, Mississauga, ON, Canada) for *in-situ* analysis. Placental PMN infiltration was studied by IHC, as described (23, 24). Rabbit anti-PMN antibody (Ab) (CLA51140, 1:100; Cedarlane Lab, ON, Canada) and mouse horseradish peroxidase (HRP)- conjugated anti-rabbit (sc-2357, 1:100, Santa Cruz Biotechnology, TX, US) were used as the primary and secondary Ab, respectively, to identify PMN in placental tissues. Diaminobenzidine molecule (DAB) (Roche, Indianapolis, IN, US) was used to detect IHC staining. Tissues were counterstained with hematoxylin. An additional set of slides was processed similarly without each primary Ab as a negative control.

### Image Analysis and Quantification

A NanoZoomer Digital Pathology (NDP) scanner (NanoZoomer 2.0RS, Hamamatsu Photonics) was used to scan slides. As previously described, densities were measured in the three different compartments (decidua, junctional zone, and labyrinth) of placentas as previously described (23). Briefly, PMN cells were counted in five fields in the labyrinth, five in the junctional zone, and three in the decidua. PMN cell densities were determined by dividing the counted number of PMNs by the field area. The mean value of PMN cell density per placental compartment was used in the statistical analysis.

### Placental Protein Extraction and Cytokine Quantification

Pierce BCA protein kit (Thermo Scientific, ON, Canada) was used to extract total protein from placentas. Bradford Protein Assay (Bio-Rad, ON, Canada) was used to quantify the whole isolated proteins. The protein suspension was aliquoted in 120 μl and kept at -80°C until analysis. Following the manufacturer’s instructions, pro-inflammatory cytokines were quantified using rat ELISA kits for IL-1β, IL-6, tumor necrosis factor (TNF-α), and Chemokine (C-X-C motif) ligand 1 (CXCL1) (R&D System, MN, US). ELISA results for placentas were calculated with protein concentration per individual sample. Fetal sera from males and females were pooled by sex within the same litter before performing ELISA assays, as only a limited amount of serum could be obtained per fetus.

### Behavioral Tests

For each subject in the study, a 10-day no-testing period was respected between subsequent behavioral experiments. All behavioral experiments were performed between 8 and 11 AM (i.e., during the daylight schedule).

Nest-seeking behavior is an olfactory discrimination test mediated by olfactory cues from the home cage bedding, and a recognized test to assess social-odor recognition of pups to find their mother (41). At P9, rodents rely on their olfactory system to communicate and discover the surrounding environment. Nest-seeking is a commonly used test to measure social-communication skills, maternal attachment and sensory integration, whose defects are key symptoms of ASD (42, 43). The test was performed as described (27).

The Open field test was performed to assess spontaneous locomotor activity in a novel environment, as described (27, 44). Briefly, the pup was placed in a corner, facing the center of the open field apparatus, and was allowed to explore for 5 min. Tracking and recording of locomotor activities started as soon as the animal was placed in the apparatus (Any-Maze Video Tracking System, Stoelting Co, Wood Dale, IL, USA). Different parameters were analyzed; namely, the total distance travelled, and duration of mobility.

### Statistical Analysis

The mean of two to three males and three females fetuses or pups per treated dam was used as n = 1 per sex and per litter to prevent the artificial samples size increase caused by treating each rat from the same litter as an independent subject. All data were collected from four individual experiments. Statistical analysis and figure representation were performed using GraphPad Prism software version 8 (San Diego, CA, USA). Shapiro-Wilk test was performed in order to assess the normality of the data. Data were normally distributed when the p-value was superior to 0.05. One-way ANOVA with Dunnett’s multiple comparisons or Kruskal-Wallis test with Dunn’s multiple comparisons were performed when samples were normally or non-normally distributed, respectively, with treatment as the fixed factor. Outliers were defined and removed by Grubbs’s test. The level of significance was set at *p < 0.05 or **p < 0.01 for all parameters. Data are presented as the adjusted mean ± standard error of the mean (SEM).

## Results

### Impact of the IL-1Ra Treatment on Maternal Weight Gain and Pro-Inflammatory Response in the Maternal Circulation

**Figure 1:**
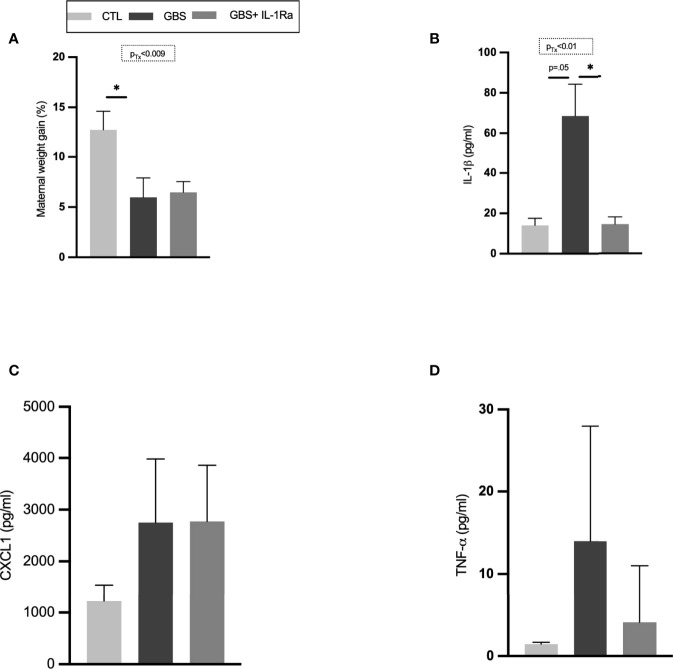
Maternal IL-1Ra treatment increased gain weight and reduced IL-1β released within the sera of GBS-infected dams. Comparison of mean maternal weight from G18 to G22 **(A)**. Mean maternal serum concentration of IL-1β **(B)**, CXCL1 **(C)** and, TNF-α **(D)** quantified by ELISA between CTL, GBS, and GBS plus IL-1Ra-exposed dams at 72 h post-GBS infection. Data were collected from three individual experiments. Analyses were performed between the experimental groups using Kruskal-Wallis test with Dunn’s multiple comparisons; *p < 0.05. Bars show mean ± SEM. Number (n) of dams: CTL (n = 6), GBS (n = 9), and GBS plus IL-1Ra (n = 8); n of serum samples: CTL (n = 4-6), GBS (n = 6-9), and GBS plus IL-1Ra (n = 6-8). CTL, control; ELISA, enzyme-linked immunosorbent assay; CXCL1, chemokine (C-X-C motif) ligand 1; G, gestational day; GBS, group B Streptococcus; IL-1Ra, IL-1 receptor antagonist; SEM, standard error of the mean.

GBS-infected dams showed decreased maternal weight gain compared to the CTL group (**Figure 1A**). No difference in maternal weight was detected between the GBS *vs* GBS plus IL-1Ra group (**Figure 1A**). IL-1Ra reduced the amount of circulating IL-1ß in the maternal sera of GBS-infected dams compared to uninfected dams (**Figure 1B**). No treatment effect was detected for the maternal levels of circulating CXCL1 and TNF-α (**Figures 1C, D****)**.

### IL-1Ra Interfered With GBS-Induced Pro-Inflammatory Response in the Placentas

**Figure 2:**
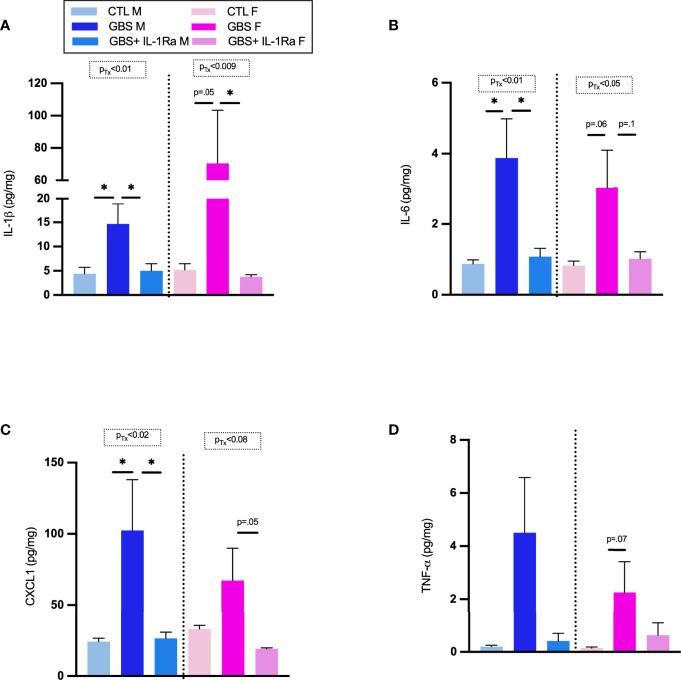
Administration of IL-1Ra to the dam decreased the levels of pro-inflammatory cytokines within GBS-infected placentas. Mean concentration of IL-1β **(A)**, IL-6 **(B)**, CXCL1 **(C)**, and TNF-α **(D)** in placentas at 72 h quantified by ELISA. Data were collected from four individual experiments. Statistical analyses were done by one-way ANOVA or Kruskal-Wallis test with Dunnett’s or Dunn’s multiple comparisons multiple between the experimental groups (CTL, GBS, and GBS plus IL-1Ra); *p < 0.05. Bars show mean ± SEM. Number (n) of male and female placentas: CTL (male and female, n = 3-6), GBS (male and female, n = 4-7), and GBS plus IL-1Ra, (male and female, n = 6-7). CXCL1, chemokine (C-X-C motif) ligand 1; ELISA, enzyme-linked immunosorbent assay; F, female; GBS, group B *Streptococcus*; IL, interleukin; IL-1Ra, IL-1 receptor antagonist; M, male; SEM, standard error of the mean; Tx, treatment.

IL-1Ra significantly decreased the levels of IL-1ß in placentas associated with male and female fetuses *in utero*-exposed to GBS (**Figure 2A**). IL-1Ra also significantly decreased IL-6 titers in male placentas and showed trend toward reduction in female placentas *in utero*-exposed to GBS (**Figure 2B**). There was a significant decrease in CXCL1 concentrations in GBS plus IL-1Ra- compared to GBS-exposed male placentas; a trend toward such a decrease was detected in placentas associated with female fetuses (**Figure 2C**). IL-1Ra did not significantly affect the concentration of TNF-α in placentas *in utero*-exposed to GBS (**Figure 2D****)**.

### Effects of IL-1Ra Treatment on PMN Infiltration in GBS-Infected Placentas

**Figure 3:**
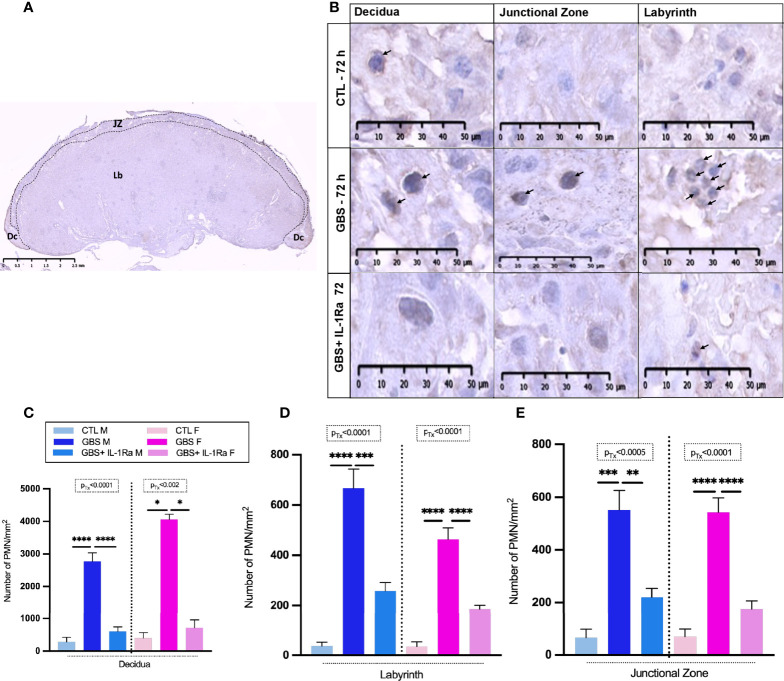
Administration of IL-1Ra to the dam alleviated PMN infiltration in GBS-infected placentas. Coronal section of a rat CTL placenta stained by IHC using PMNs Ab, black dashed lines define the placental compartments [decidua (Dc), junctional zone (JZ), and labyrinth (Lb)] **(A)**. Representative images of PMN (arrows) infiltrates in the Dc, JZ, and Lb of placentas at 72 h post-GBS inoculation **(B)**. Mean density (n of field = 3-5/compartment, field unit = mm2) of PMNs identified by IHC in the Dc **(C)**, Lb **(D)**, and Jz **(E)**. Data were collected from four individual experiments. Statistical analyses were performed by one-way ANOVA or Kruskal-Wallis test with Dunnett’s or Dunn’s multiple comparisons multiple between the experimental groups (CTL, GBS, and GBS plus IL-1Ra); *p < 0.05, **p < 0.01, ***p ≤ 0.001, ****p ≤ 0.0001. Bars show mean ± SEM. Number (n) of male and female placentas: CTL [male (n = 3); female (n = 3)], GBS [male (n = 5); female (n = 5)] and, GBS plus IL-1Ra [male (n = 6), female (n = 6)]. Ab, antibody; Dc, decidua; F, female; GBS, group B Streptococcus; IL-1Ra, IL-1 receptor antagonist; Jz: junctional zone; Lb: labyrinth; M, male; n, number; PMN, polymorphonuclear cell; SEM, standard error of the mean Tx, treatment.

IL-1Ra treatment reduced the PMN cell density in all GBS-infected placental compartments (**Figures 3A, B**) of both sexes, namely the decidua (**Figure 3C**), labyrinth (**Figure 3D**) and junctional zone (**Figure 3E**).

### IL-1Ra Reduced the Levels of Circulating IL-1ß in GBS-Exposed Fetuses

**Figure 4:**
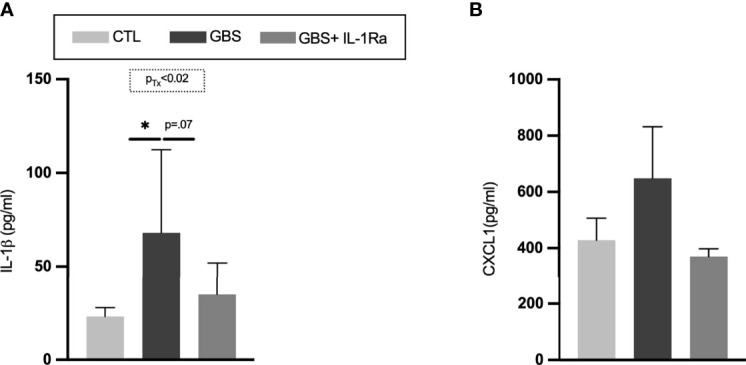
Administration of IL-1Ra to the dam reduced the levels of IL-1β in fetal sera. Mean titres of IL-1β **(A)**, and CXCL1 **(B)** in circulating fetal sera. Data were collected from four individual experiments. Analyses were done by one-way ANOVA test between the experimental groups (CTL, GBS, and GBS plus IL-1Ra) when treatment was significant; *p < 0.05. Number (n) of male and female fetuses: CTL (n = 4-6), GBS (n = 5-7), and GBS plus IL-1Ra (n = 5-7). Bars show mean ± SEM. CTL, control; CXCL1, chemokine (C-X-C motif) ligand 1; GBS, group B *Streptococcus*; IL-1, interleukin-1; IL-1Ra, IL-1 receptor antagonist; SEM, standard error of the mean; Tx, treatment.

GBS-induced chorioamnionitis significantly increased the level of circulating IL-1ß in fetal sera compared to saline-exposed subjects (**Figure 4A**). The IL-1Ra treatment induced a trend toward a reduction of IL-1ß titers in the fetal sera in GBS-exposed fetuses (**Figure 4A**). No difference was detected in the levels of CXCL1 in the fetal sera between experimental conditions (**Figure 4B**).

### No Adverse Effect of IL-1Ra Was Observed on Placental and Fetal Weight, Maternofetal Infection Sickness Behavior and Fetal Mortality

**Figure 5:**
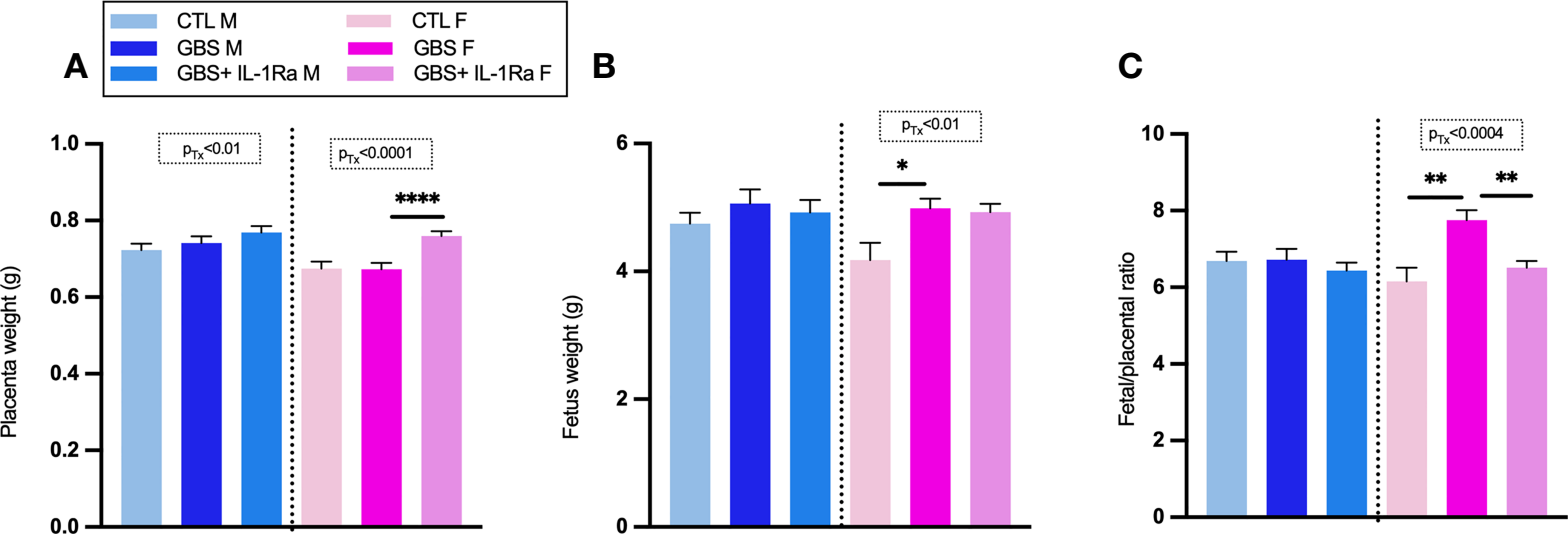
Impact of IL-1Ra on fetal and placental weight in response to GBS infection. IL-1Ra positively controls the fetal and placental weight in response to GBS infection. Mean of placental weight **(A)**, fetus weight **(B)** and the ratio of fetus placenta at G22 **(C)**. Number (n) of placentas: CTL (male [n = 43), female (n = 23)], GBS [male (n = 38), female (n = 43)], and GBS plus IL-1Ra [male (n = 33), female (n = 32)]. Data were collected from four individual experiments. Analyses were done by ANOVA test separately for males and females with Dunnett’s multiple comparisons between groups (CTL, GBS, and GBS plus IL-1Ra) when treatment was significant. *p < 0.05, **p < 01, ****p ≤ 0.001 Bars show mean ± SEM. F, female; G, gestational day; GBS, group B Streptococcus; IL-1Ra, IL-1 receptor antagonist; M, male; SEM, standard error of the mean.

**Figure 6:**
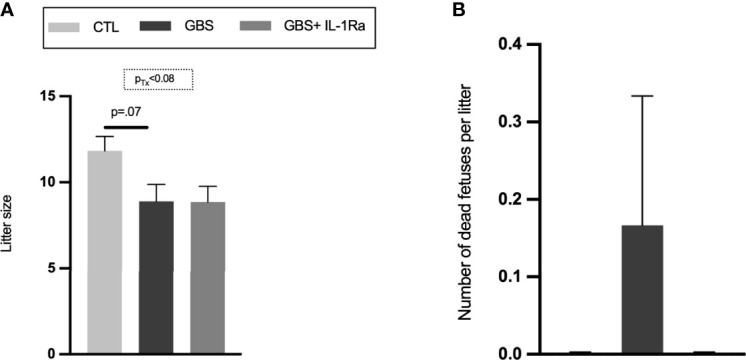
Litter size and fetal mortality. Mean number of alive fetuses per litter **(A)**, percentage of dead fetuses per litter **(B)**. Number (n) of litters: CTL (n = 6), GBS (n = 9) and, GBS plus IL-1Ra (n = 7). Data were collected from four individual experiments. Analyses were done by ANOVA test separately for males and females. Dunnett’s multiple comparisons were done between groups (CTL, GBS, and GBS plus IL-1Ra). Bars show mean ± SEM. GBS, group B *Streptococcus*; IL-1Ra, IL-1 receptor antagonist; n, number; SEM, standard error of the mean.

Modest but significant variations of placental, fetal weight and fetal/placental ratio were observed in female placental and fetal tissues from the GBS plus IL-1Ra group compared to the GBS group (**Figures 5A–C****)**. In females only, IL-Ra brought back fetal/placental ratio to CTL level (**Figure 5C**). There was no increased mortality, no difference in sickness-related behavior, and no premature delivery observed between the three experimental conditions, showing that IL-1 blockade did not aggravate GBS infection in dams (data not shown). No change in the maternal weight gain was observed in GBS plus IL-1Ra compared to the GBS dams (**Figure 1A****)**. Litter size was identical between GBS plus IL-1Ra- *versus* GBS-exposed dams (**Figure 6A**). The fetal mortality rate was identical between GBS *versus* GBS plus IL-1Ra groups (**Figure 6B**).

### Effect of IL-1Ra on Behavioral Tests

**Figure 7:**
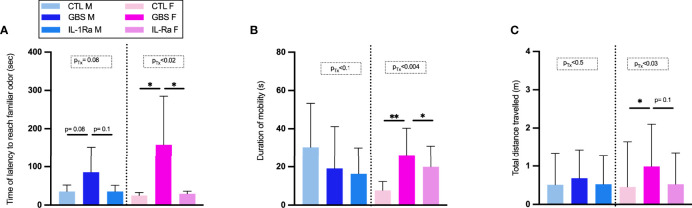
Effect of IL-1Ra on reducing neurobehavioral impairments in male and female offspring. Time of latency to reach familiar odor **(A)**, Duration of mobility **(B)**, Total distance travelled **(C)**. Data were collected from three individual experiments. Statistical analyses were done by one-way ANOVA or Kruskal-Wallis test with Dunnett’s or Dunn’s multiple comparisons multiple between the experimental groups (CTL, GBS, and GBS plus IL-1Ra); *p < 0.05, **p < 01. Bars show mean ± SEM. Number (n) of male and female: CTL (male and female, n = 6-11), GBS (male and female, n = 7- 10), and GBS plus IL-1Ra, (male and female, n = 4-8). Bars show mean ± SEM. F, female; GBS, group B Streptococcus; IL, interleukin; IL-1Ra, IL-1 receptor antagonist; M, male; n, number; SEM, standard error of the mean; Tx, treatment.

In the nest-seeking test at P9, our results showed that the concomitant *in utero* exposure to GBS infection with IL-1Ra prevented the subsequent increase in the latency to reach the familiar odor observed in GBS-exposed male as well as female offspring (**Figure 7A**). In the open field test at P20, our results showed that the concomitant *in utero* exposure to GBS infection with IL-1Ra prevented the increased duration of mobility and total distance travelled in female, but not male, offspring *in utero*-exposed to GBS (**Figures 7B, C**).

## Discussion

### Key Findings

This study aimed to test the effect of end-gestational IL-1 blockade in the context of GBS-induced chorioamnionitis. The IL-1 blockade has been shown to be both placento- and feto-protective in an MIA model using the LPS from *Escherichia coli* (40, 45–47). However, no study has yet investigated whether such intervention would also be effective in chorioamnionitis triggered by GBS. Our results demonstrated the key role of IL-1 in driving GBS-induced chorioamnionitis through *(i)* upregulating pro-inflammatory cytokines and chemokines, *(ii)* increasing PMN infiltration in the decidua and labyrinth, *(iii)* inducing a FIRS within the labyrinth as well as downstream within the fetal sera, and (v) reducing neurobehavioral impairments in male and female offspring. Finally, on IL-1Ra, GBS infection remained self-limited, and infected dams did not present any sign of infectious aggravation compared to sole GBS infection.

### Previous Studies

Preclinical studies of GBS-induced sepsis showed that GBS-driven inflammation was mainly driven by TLR2/6 and β-hemolysin/ (NLR)-P3 pathways and IL-1 (13–16). Pathogen-induced placental inflammation was previously addressed with non-GBS models of MIA, using mainly inactivated bacteria and pathogen components involved in chorioamnionitis mainly *via* ligands/receptors such as LPS/TLR4 (12, 48–52). Our findings are consistent with the placento-protective efficacy and safety of IL-1 blockade as shown in other preclinical models using LPS from *E. coli* (TLR4 agonist), lipoteichoic acid or Pam3CysSerLys4 (TLR2 agonists) as inducers of MIA in pregnant mice, rat, rhesus macaque and sheep models (5, 8, 9, 53). This study demonstrated benefits against the pro-inflammatory immune response triggered by GBS-induced chorioamnionitis and showed that hrIL-1Ra was well-tolerated in a model of end-gestational infection. This preclinical study, added to previous research using other pathogen components as immune triggers during gestation, would bring us to the threshold of assessing the feasibility of a phase 2 randomized controlled trial as a repurpose of an already FDA-approved agent (28, 46).

Clinical trials of anti-inflammatory interventions in the hope to protect maternofetal organs - especially the fetal brain - against pathogen-induced inflammatory responses have been disappointing mainly because of their lack of efficacy (54, 55). Newborns from mothers at high risk of infections did not show better neurobehavioral outcomes when treated *in utero* by sole antibiotic administration (56, 57). Administration of antibiotics before preterm birth did not alleviate some unfavourable neurobehavioral outcomes but increased by two-fold the occurrence of CP (54). The adverse effects of antibiotics might result from possible exacerbation of pathogen-induced inflammatory responses as observed in the same preclinical model of end-gestational GBS infection, in which there was an antibiotic-induced release of pro-inflammatory components (58). Trials of administration of corticosteroids to pregnant mothers or postnatally to newborns showed that corticosteroids do not improve some neurobehavioral outcomes and even result in adverse effects in specific therapeutic designs (59–62). A recent phase 3 randomized and placebo-controlled trial assessed the efficacy of erythropoietin on mortality and neurological sequelae of preterm newborns; results did not show any difference between treated *versus* placebo groups (63). These disappointing outcomes support the need to investigate the potential benefit of more targeted immunomodulators, such as IL-1Ra, to find a better compromise between the benefits and risks of drugs used during the perinatal period. In this regard, we chose to test the role of *in utero* blockade of IL-1 during GBS infection of dams on the nest-seeking behavior of the offspring. The nest-seeking test evaluates impaired communication skills, maternal attachment and sensory integration in pups, which are recognized autistic-like traits in rodent models. Previous works showed that male, but not female, pups *in utero* exposed to GBS develop autistic-like traits, and among them an increased latency in finding the familiar odor of their nest (23). Our experiments show that IL-1 has a key role in such FIRS-induced autistic-like traits. Previous works showed that *in utero* exposure to GBS triggered a hyperactive-like behavior (23) in female offspring. Interestingly, our results show that IL-1 is also at play in the FIRS-induced hyperactive behavior in female offspring.

### Limitations

In this novel preclinical research, we showed the benefit of maternal human recombinant IL-1Ra intervention to control the GBS-induced MIA. However, our work presents some limitations. *Firstly*, we did not address on which tissue beyond the placenta and cell type(s) (*e.g.* maternal *versus* fetal white blood cells, white blood cell type, or placental cell) the IL-1 blockade exerts its effect and in which extent IL-1Ra crosses the placental barrier in the context of GBS infection. These important mechanistic aspects will remain to be investigated. *Secondly*, our study showed sex-specific differences in some impacts of the IL-1Ra treatment, but we did not address the mechanism underpinning it. For instance, sex hormones such as androgen peaking in rat fetuses at the end of gestation, estrogen, or glucocorticoid, which have been reported to be differentially expressed in male *versus* female placenta and other developing organs, might play a role in the sex differences we noticed (64, 65). However, the complex study of such possible involvement of hormones in regulating the sex-dichotomic developmental inflammatory response is beyond the goal of the present study and will be addressed in subsequent research. *Thirdly*, beyond the brain, the impact of IL-1 blockade on the prevention of the MIA-induced fetal multiorgan - *e.g.* bronchopulmonary dysplasia, retinopathy, necrotizing enterocolitis - defects remains another essential subsequent research topic.

### Future Directions

Maternal hr IL-1Ra therapy, using doses already recommended for human inflammatory diseases, may meet the safety/efficacy criteria to launch a phase II placento- and neuro-protective clinical trial (37, 66). However, the translation from animals to humans requires caution as cytokines are involved in many neurodevelopmental mechanisms and other physiological functions (67).

## Data Availability Statement

The raw data supporting the conclusions of this article will be made available by the authors, without undue reservation.

## Ethics Statement

The animal study was reviewed and approved by Research Institute of McGill University Hospital, McGill University.

## Author Contributions

TA contributed to writing the manuscript, performing experiments, data analysis, and statistical analysis. SV contributed to writing the manuscript. GS contributed to the conception and design the study. M-JA contributed to performing experiments, conception and design of the study. All authors contributed to manuscript writing, revision, read, and approved the submitted version.

## Funding

This work was supported by a grant from the Canadian Institute of Health Research (CIHR operating grant #6847), SV had scholarships from the Program of Pharmacology, McGill University, and from the Research Institute of McGill University Health Center (RI MUHC).

## Conflict of Interest

The authors declare that the research was conducted in the absence of any commercial or financial relationships that could be construed as a potential conflict of interest.

## Publisher’s Note

All claims expressed in this article are solely those of the authors and do not necessarily represent those of their affiliated organizations, or those of the publisher, the editors and the reviewers. Any product that may be evaluated in this article, or claim that may be made by its manufacturer, is not guaranteed or endorsed by the publisher.

## Abbreviations

Ab, antibody; ADHD, Attention deficit hyperactivity disorder; ASD, Autism spectrum disorder; BHI, Brain heart infusion; CP; cerebral palsy; CTL, Control; CXCL, Chemokine (C-X-C motif) ligand; Dc, Decidua; F, Female; FIRS, Fetal inflammatory response syndrome; GBS, Group B *Streptococcus;* hr, Human recombinant; IHC, Immunohistochemistry; IL, Interleukin; IL-1Ra, Interleukin-1 receptor antagonist; i.p., Intraperitoneally; JZ, Junctional zone; L, Labyrinth; LPS, lipopolysaccharide; M, Male; N, Number; MIA, Maternal immune activation; NLR, Nod-like receptor; PMN, Polymorphonuclear cells; Sec, Second; SEM, Standard error of the mean; TLR, Toll like receptor; TNF, Tumor necrosis factor; Tx, Treatment.

## References

[1] Limperopoulos C Bassan H Sullivan NR Soul JS Robertson RL Jr. Moore M. Positive Screening for Autism in Ex-Preterm Infants: Prevalence and Risk Factors. Pediatrics (2008) 121(4):758–65. doi: 10.1542/peds.2007-2158 PMC2703587 18381541

[2] Nelson KB Chang T. Is Cerebral Palsy Preventable? Curr Opin Neurol (2008) 21(2):129–35. doi: 10.1097/WCO.0b013e3282f4958b 18317269

[3] Deverman BE Patterson PH. Cytokines and CNS Development. Neuron (2009) 64(1):61–78. doi: 10.1016/j.neuron.2009.09.002 19840550

[4] Meyer U Feldon J Fatemi SH. *In-Vivo* Rodent Models for the Experimental Investigation of Prenatal Immune Activation Effects in Neurodevelopmental Brain Disorders. Neurosci Biobehav Rev (2009) 33(7):1061–79. doi: 10.1016/j.neubiorev.2009.05.001 19442688

[5] Nadeau-Vallee M Quiniou C Palacios J Hou X Erfani A Madaan A. Novel Noncompetitive IL-1 Receptor-Biased Ligand Prevents Infection- and Inflammation-Induced Preterm Birth. J Immunol (2015) 195(7):3402–15. doi: 10.4049/jimmunol.1500758 26304990

[6] Ornoy A Weinstein-Fudim L Ergaz Z. Prenatal Factors Associated With Autism Spectrum Disorder (ASD). Reprod Toxicol (2015) 56:155–69. doi: 10.1016/j.reprotox.2015.05.007 26021712

[7] Nadeau-Vallee M Chin PY Belarbi L Brien ME Pundir S Berryer MH. Antenatal Suppression of IL-1 Protects Against Inflammation-Induced Fetal Injury and Improves Neonatal and Developmental Outcomes in Mice. J Immunol (2017) 198(5):2047–62. doi: 10.4049/jimmunol.1601600 28148737

[8] Girard S Tremblay L Lepage M Sebire G. IL-1 Receptor Antagonist Protects Against Placental and Neurodevelopmental Defects Induced by Maternal Inflammation. J Immunol (2010) 184(7):3997–4005. doi: 10.4049/jimmunol.0903349 20181892

[9] Malkova NV Yu CZ Hsiao EY Moore MJ Patterson PH. Maternal Immune Activation Yields Offspring Displaying Mouse Versions of the Three Core Symptoms of Autism. Brain Behav Immun (2012) 26(4):607–16. doi: 10.1016/j.bbi.2012.01.011 PMC3322300 22310922

[10] Baharnoori M Bhardwaj SK Srivastava LK. Effect of Maternal Lipopolysaccharide Administration on the Development of Dopaminergic Receptors and Transporter in the Rat Offspring. PloS One (2013) 8(1):e54439. doi: 10.1371/journal.pone.0054439 23349891 PMC3547943

[11] Fernandez de Cossio L Guzman A van der Veldt S Luheshi GN. Prenatal Infection Leads to ASD-Like Behavior and Altered Synaptic Pruning in the Mouse Offspring. Brain Behav Immun (2017) 63:88–98. doi: 10.1016/j.bbi.2016.09.028 27697456

[12] Hui CW St-Pierre A El Hajj H Remy Y Hebert SS Luheshi GN. Prenatal Immune Challenge in Mice Leads to Partly Sex-Dependent Behavioral, Microglial, and Molecular Abnormalities Associated With Schizophrenia. Front Mol Neurosci (2018) 11:13. doi: 10.3389/fnmol.2018.00013 29472840 PMC5809492

[13] Henneke P Dramsi S Mancuso G Chraibi K Pellegrini E Theilacker C. Lipoproteins Are Critical TLR2 Activating Toxins in Group B Streptococcal Sepsis. J Immunol (2008) 180(9):6149–58. doi: 10.4049/jimmunol.180.9.6149 18424736

[14] Costa A Gupta R Signorino G Malara A Cardile F Biondo C. Activation of the NLRP3 Inflammasome by Group B Streptococci. J Immunol (2012) 188(4):1953–60. doi: 10.4049/jimmunol.1102543 PMC3273589 22250086

[15] Gupta R Ghosh S Monks B DeOliveira RB Tzeng TC Kalantari P. RNA and Beta-Hemolysin of Group B Streptococcus Induce Interleukin-1beta (IL-1beta) by Activating NLRP3 Inflammasomes in Mouse Macrophages. J Biol Chem (2014) 289(20):13701–5. doi: 10.1074/jbc.C114.548982 PMC4022842 24692555

[16] Patras KA Nizet V. Group B Streptococcal Maternal Colonization and Neonatal Disease: Molecular Mechanisms and Preventative Approaches. Front Pediatr (2018) 6:27. doi: 10.3389/fped.2018.00027 29520354 PMC5827363

[17] Allard MJ Brochu ME Bergeron JD Sebire G. Hyperactive Behavior in Female Rats *In Utero*-Exposed to Group B Streptococcus-Induced Inflammation. Int J Dev Neurosci (2018) 69:17–22. doi: 10.1016/j.ijdevneu.2018.06.005 29920305

[18] Allard MJ Brochu ME Bergeron JD Segura M Sebire G. Causal Role of Group B Streptococcus-Induced Acute Chorioamnionitis in Intrauterine Growth Retardation and Cerebral Palsy-Like Impairments. J Dev Orig Health Dis (2019) 10(5):595–602. doi: 10.1017/S2040174418001083 30626456

[19] Mann JR McDermott S. Are Maternal Genitourinary Infection and Pre-Eclampsia Associated With ADHD in School-Aged Children? J Atten Disord (2011) 15(8):667–73. doi: 10.1177/1087054710370566 20837984

[20] Strunk T Inder T Wang X Burgner D Mallard C Levy O. Infection-Induced Inflammation and Cerebral Injury in Preterm Infants. Lancet Infect Dis (2014) 14(8):751–62. doi: 10.1016/S1473-3099(14)70710-8 PMC4125363 24877996

[21] Kim CJ Romero R Chaemsaithong P Chaiyasit N Yoon BH Kim YM. Acute Chorioamnionitis and Funisitis: Definition, Pathologic Features, and Clinical Significance. Am J Obstet Gynecol (2015) 213(4 Suppl):S29–52. doi: 10.1016/j.ajog.2015.08.040 PMC4774647 26428501

[22] Estes ML McAllister AK. Maternal Immune Activation: Implications for Neuropsychiatric Disorders. Science (2016) 353(6301):772–7. doi: 10.1126/science.aag3194 PMC5650490 27540164

[23] Allard MJ Bergeron JD Baharnoori M Srivastava LK Fortier LC Poyart C. A Sexually Dichotomous, Autistic-Like Phenotype Is Induced by Group B Streptococcus Maternofetal Immune Activation. Autism Res (2017) 10(2):233–45. doi: 10.1002/aur.1647 27220806

[24] Allard MJ Giraud A Segura M Sebire G. Sex-Specific Maternofetal Innate Immune Responses Triggered by Group B Streptococci. Sci Rep (2019) 9(1):8587. doi: 10.1038/s41598-019-45029-x 31197179 PMC6565749

[25] Biondo C Mancuso G Midiri A Signorino G Domina M Lanza Cariccio V. Essential Role of Interleukin-1 Signaling in Host Defenses Against Group B Streptococcus. mBio (2014) 5(5):e01428–01414. doi: 10.1128/mBio.01428-14 PMC4166122 25205091

[26] Bergeron J Gerges N Guiraut C Grbic D Allard MJ Fortier LC. Activation of the IL-1beta/CXCL1/MMP-10 Axis in Chorioamnionitis Induced by Inactivated Group B Streptococcus. Placenta (2016) 47:116–23. doi: 10.1016/j.placenta.2016.09.016 27780533

[27] Bergeron JD Deslauriers J Grignon S Fortier LC Lepage M Stroh T. White Matter Injury and Autistic-Like Behavior Predominantly Affecting Male Rat Offspring Exposed to Group B Streptococcal Maternal Inflammation. Dev Neurosci (2013) 35(6):504–15. doi: 10.1159/000355656 24246964

[28] Rosenzweig JM Lei J Burd I. Interleukin-1 Receptor Blockade in Perinatal Brain Injury. Front Pediatr (2014) 2:108. doi: 10.3389/fped.2014.00108 25340046 PMC4187538

[29] Accessdata.fda.gov. "Kineret® (Anakinra) for Injection, for Subcutaneous Use Initial U.S. Approval: 2001". (2001) USA, Food And Drug Administration.

[30] Perrien DS Brown EC Fletcher TW Irby DJ Aronson J Gao GG. Interleukin-1 and Tumor Necrosis Factor Antagonists Attenuate Ethanol-Induced Inhibition of Bone Formation in a Rat Model of Distraction Osteogenesis. J Pharmacol Exp Ther (2002) 303(3):904–8. doi: 10.1124/jpet.102.039636 12438508

[31] Banwell V Sena ES Macleod MR. Systematic Review and Stratified Meta-Analysis of the Efficacy of Interleukin-1 Receptor Antagonist in Animal Models of Stroke. J Stroke Cerebrovasc Dis (2009) 18(4):269–76. doi: 10.1016/j.jstrokecerebrovasdis.2008.11.009 19560680

[32] Tegtmeyer K Atassi G Zhao J Maloney NJ Lio PA. Off-Label Studies on Anakinra in Dermatology: A Review. J Dermatolog Treat (2022) 33(1):73–86. doi: 10.1080/09546634.2020.1755417 32279586

[33] Chambers CD Tutuncu ZN Johnson D Jones KL. Human Pregnancy Safety for Agents Used to Treat Rheumatoid Arthritis: Adequacy of Available Information and Strategies for Developing Post-Marketing Data. Arthritis Res Ther (2006) 8(4):215. doi: 10.1186/ar1977 16774693 PMC1779429

[34] Berger CT Recher M Steiner U Hauser TM. A Patient's Wish: Anakinra in Pregnancy. Ann Rheum Dis (2009) 68(11):1794–5. doi: 10.1136/ard.2008.105833 19822718

[35] Chang Z Spong CY Jesus AA Davis MA Plass N Stone DL. Anakinra Use During Pregnancy in Patients With Cryopyrin-Associated Periodic Syndromes (CAPS). Arthritis Rheumatol (2014) 66(11):3227–32. doi: 10.1002/art.38811 PMC4323990 25223501

[36] Youngstein T Hoffmann P Gul A Lane T Williams R Rowczenio DM. International Multi-Centre Study of Pregnancy Outcomes With Interleukin-1 Inhibitors. Rheumatol (Oxford) (2017) 56(12):2102–8. doi: 10.1093/rheumatology/kex305 PMC6251516 28968868

[37] Brien ME Gaudreault V Hughes K Hayes DJL Heazell AEP Girard S. A Systematic Review of the Safety of Blocking the IL-1 System in Human Pregnancy. J Clin Med (2021) 11(1):225. doi: 10.3390/jcm11010225 35011965 PMC8745599

[38] Karakas O Erden A Unlu S Erol SA Goncu Ayhan S Ozdemir B. Can Anakinra and Corticosteroid Treatment Be an Effective Option in Pregnant Women With Severe Covid-19? Women Health (2021) 61(9):872–9. doi: 10.1080/03630242.2021.1981517 PMC8477586 34551674

[39] Kyriazopoulou E Poulakou G Milionis H Metallidis S Adamis G Tsiakos K. Early Treatment of COVID-19 With Anakinra Guided by Soluble Urokinase Plasminogen Receptor Plasma Levels: A Double-Blind, Randomized Controlled Phase 3 Trial. Nat Med (2021) 27(10):1752–60. doi: 10.1038/s41591-021-01499-z PMC8516650 34480127

[40] Girard S Sebire G Kadhim H. Proinflammatory Orientation of the Interleukin 1 System and Downstream Induction of Matrix Metalloproteinase 9 in the Pathophysiology of Human Perinatal White Matter Damage. J Neuropathol Exp Neurol (2010) 69(11):1116–29. doi: 10.1097/NEN.0b013e3181f971e4 20940629

[41] Gregory EH Pfaff DW. Development of Olfactory-Guided Behavior in Infant Rats. Physiol Behav (1971) 6(5):573–6. doi: 10.1016/0031-9384(71)90208-3 5149448

[42] Schneider T Przewlocki R. Behavioral Alterations in Rats Prenatally Exposed to Valproic Acid: Animal Model of Autism. Neuropsychopharmacology (2005) 30(1):80–9. doi: 10.1038/sj.npp.1300518 15238991

[43] Favre MR Barkat TR Lamendola D Khazen G Markram H Markram K. General Developmental Health in the VPA-Rat Model of Autism. Front Behav Neurosci (2013) 7:88. doi: 10.3389/fnbeh.2013.00088 23898245 PMC3721005

[44] Baharnoori M Bhardwaj SK Srivastava LK. Neonatal Behavioral Changes in Rats With Gestational Exposure to Lipopolysaccharide: A Prenatal Infection Model for Developmental Neuropsychiatric Disorders. Schizophr Bull (2012) 38(3):444–56. doi: 10.1093/schbul/sbq098 PMC3329978 20805287

[45] Girard S Kadhim H Larouche A Roy M Gobeil F Sebire G. Pro-Inflammatory Disequilibrium of the IL-1 Beta/IL-1ra Ratio in an Experimental Model of Perinatal Brain Damages Induced by Lipopolysaccharide and Hypoxia-Ischemia. Cytokine (2008) 43(1):54–62. doi: 10.1016/j.cyto.2008.04.007 18511291

[46] Brochu ME Girard S Lavoie K Sebire G. Developmental Regulation of the Neuroinflammatory Responses to LPS and/or Hypoxia-Ischemia Between Preterm and Term Neonates: An Experimental Study. J Neuroinflamm (2011) 8:55. doi: 10.1186/1742-2094-8-55 PMC3121616 21599903

[47] Deslauriers J Lefrancois M Larouche A Sarret P Grignon S. Antipsychotic-Induced DRD2 Upregulation and Its Prevention by Alpha-Lipoic Acid in SH-SY5Y Neuroblastoma Cells. Synapse (2011) 65(4):321–31. doi: 10.1002/syn.20851 20730801

[48] Ashdown H Poole S Boksa P Luheshi GN. Interleukin-1 Receptor Antagonist as a Modulator of Gender Differences in the Febrile Response to Lipopolysaccharide in Rats. Am J Physiol Regul Integr Comp Physiol (2007) 292(4):R1667–1674. doi: 10.1152/ajpregu.00274.2006 17138728

[49] Foley KA MacFabe DF Vaz A Ossenkopp KP Kavaliers M. Sexually Dimorphic Effects of Prenatal Exposure to Propionic Acid and Lipopolysaccharide on Social Behavior in Neonatal, Adolescent, and Adult Rats: Implications for Autism Spectrum Disorders. Int J Dev Neurosci (2014) 39:68–78. doi: 10.1016/j.ijdevneu.2014.04.001 24747144

[50] Schaafsma SM Gagnidze K Reyes A Norstedt N Mansson K Francis K. Sex-Specific Gene-Environment Interactions Underlying ASD-Like Behaviors. Proc Natl Acad Sci USA (2017) 114(6):1383–8. doi: 10.1073/pnas.1619312114 PMC5307430 28115688

[51] Andrade EB Magalhaes A Puga A Costa M Bravo J Portugal CC. A Mouse Model Reproducing the Pathophysiology of Neonatal Group B Streptococcal Infection. Nat Commun (2018) 9(1):3138. doi: 10.1038/s41467-018-05492-y 30087335 PMC6081475

[52] Custodio CS Mello BSF Filho A de Carvalho Lima CN Cordeiro RC Miyajima F. Neonatal Immune Challenge With Lipopolysaccharide Triggers Long-Lasting Sex- and Age-Related Behavioral and Immune/Neurotrophic Alterations in Mice: Relevance to Autism Spectrum Disorders. Mol Neurobiol (2018) 55(5):3775–88. doi: 10.1007/s12035-017-0616-1 28536974

[53] Kallapur SG Nitsos I Moss TJ Polglase GR Pillow JJ Cheah FC. IL-1 Mediates Pulmonary and Systemic Inflammatory Responses to Chorioamnionitis Induced by Lipopolysaccharide. Am J Respir Crit Care Med (2009) 179(10):955–61. doi: 10.1164/rccm.200811-1728OC PMC2684020 19234101

[54] Kenyon S Pike K Jones DR Brocklehurst P Marlow N Salt A. Childhood Outcomes After Prescription of Antibiotics to Pregnant Women With Spontaneous Preterm Labour: 7-Year Follow-Up of the ORACLE II Trial. Lancet (2008) 372(9646):1319–27. doi: 10.1016/S0140-6736(08)61203-9 18804276

[55] Shepherd E Salam RA Middleton P Makrides M McIntyre S Badawi N. Antenatal and Intrapartum Interventions for Preventing Cerebral Palsy: An Overview of Cochrane Systematic Reviews. Cochrane Database Syst Rev (2017) 8:CD012077. doi: 10.1002/14651858.CD012077.pub2 28786098 PMC6483544

[56] Lin FY Brenner RA Johnson YR Azimi PH Philips JB Regan JA. The Effectiveness of Risk-Based Intrapartum Chemoprophylaxis for the Prevention of Early-Onset Neonatal Group B Streptococcal Disease. Am J Obstet Gynecol (2001) 184(6):1204–10. doi: 10.1067/mob.2001.113875 11349189

[57] Ahmadzia HK Heine RP. Diagnosis and Management of Group B Streptococcus in Pregnancy. Obstet Gynecol Clin North Am (2014) 41(4):629–47. doi: 10.1016/j.ogc.2014.08.009 25454995

[58] Giraud A Allard MJ Segura M Roche F Patural H Sebire G. Ampicillin Treatment Increases Placental Interleukin-1 Beta Concentration and Polymorphonuclear Infiltration in Group B Streptococcus-Induced Chorioamnionitis: A Preclinical Study. Neonatology (2020) 117(3):369–73. doi: 10.1159/000506906 32375156

[59] Mesples B Plaisant F Gressens P. Effects of Interleukin-10 on Neonatal Excitotoxic Brain Lesions in Mice. Brain Res Dev Brain Res (2003) 141(1-2):25–32. doi: 10.1016/s0165-3806(02)00636-3 12644245

[60] Rodts-Palenik S Wyatt-Ashmead J Pang Y Thigpen B Cai Z Rhodes P. Maternal Infection-Induced White Matter Injury Is Reduced by Treatment With Interleukin-10. Am J Obstet Gynecol (2004) 191(4):1387–92. doi: 10.1016/j.ajog.2004.06.093 15507970

[61] Lyng K Munkeby BH Saugstad OD Stray-Pedersen B Froen JF. Effect of Interleukin-10 on Newborn Piglet Brain Following Hypoxia-Ischemia and Endotoxin-Induced Inflammation. Biol Neonate (2005) 87(3):207–16. doi: 10.1159/000083131 15637455

[62] Halliday HL Ehrenkranz RA Doyle LW. Early (< 8 Days) Postnatal Corticosteroids for Preventing Chronic Lung Disease in Preterm Infants. Cochrane Database Syst Rev (2009) 1):CD001146. doi: 10.1002/14651858.CD001146.pub2 19160190

[63] Juul SE Comstock BA Wadhawan R Mayock DE Courtney SE Robinson T. A Randomized Trial of Erythropoietin for Neuroprotection in Preterm Infants. N Engl J Med (2020) 382(3):233–43. doi: 10.1056/NEJMoa1907423 PMC7060076 31940698

[64] Mueller BR Bale TL. Sex-Specific Programming of Offspring Emotionality After Stress Early in Pregnancy. J Neurosci (2008) 28(36):9055–65. doi: 10.1523/JNEUROSCI.1424-08.2008 PMC2731562 18768700

[65] Saif Z Hodyl NA Stark MJ Fuller PJ Cole T Lu N. Expression of Eight Glucocorticoid Receptor Isoforms in the Human Preterm Placenta Vary With Fetal Sex and Birthweight. Placenta (2015) 36(7):723–30. doi: 10.1016/j.placenta.2015.05.001 PMC4830346 25990415

[66] Church LD Cook GP McDermott MF. Primer: Inflammasomes and Interleukin 1beta in Inflammatory Disorders. Nat Clin Pract Rheumatol (2008) 4(1):34–42. doi: 10.1038/ncprheum0681 18172447

[67] Mehler MF Kessler JA. Cytokines in Brain Development and Function. Adv Protein Chem (1998) 52:223–51. doi: 10.1016/s0065-3233(08)60437-4 9917922

